# What Doctors Wear Matters: Insights From Visitors at Riyadh's Primary Healthcare Centers

**DOI:** 10.7759/cureus.113363

**Published:** 2026-07-25

**Authors:** Hamad A Alhussain, Abdullah A Alajlan, Omar Alghamdi, Abdulazia S Alhodairy

**Affiliations:** 1 Family Medicine, Family Medicine Academy at Riyadh Second Health Cluster, Riyadh, SAU; 2 Family Medicine, King Fahad Medical City, Riyadh, SAU; 3 College of Medicine, Majmaah University, Riyadh, SAU

**Keywords:** cultural values, doctor–patient relationship, dress code, patient perception, physician attire, primary healthcare, saudi arabia, white coat

## Abstract

Background: How a physician dresses has been known, since the time of Hippocrates, to shape the way patients see and relate to their doctor. In a country like Saudi Arabia, where cultural norms, religious values, and medical professionalism intersect in distinctive ways, the question of what physicians ought to wear is more than a trivial matter of aesthetics. Despite a growing literature on this subject, relatively little research has focused specifically on primary healthcare settings within the Gulf region, where the majority of routine patient-physician encounters take place.

Objectives: This study set out to examine how patients attending primary healthcare centers (PHCs) affiliated with the Riyadh Second Health Cluster perceive and rate different physician attire options, to gauge the overall importance they place on appearance, and to explore how attire-related perceptions translate into attitudes toward the doctor-patient relationship.

Methods: A cross-sectional survey was conducted from the 1st of April to the 31st of May, 2026. A self-administered bilingual (Arabic/English) questionnaire, adapted from validated tools in the literature and reviewed by two family medicine consultants, was completed by 1,176 adult PHC visitors aged 14 years and above. Participants rated male and female physician attire items and 11 relationship/perception outcomes on a five-point Likert scale. Chi-square tests, Mann-Whitney U tests, and Kruskal-Wallis tests were applied where appropriate, with statistical significance set at p < 0.05.

Results: Participants had a mean age of 33.3 ± 9.8 years; 597 (50.8%) were male. Eight in 10 participants (950; 80.8%; 95% CI: 78.5-83.0%) considered physician appearance important, a finding that held across all demographic subgroups. The white medical lab coat topped the preference list for both male (1005; 85.5%; 95% CI: 83.4-87.5%) and female (922; 78.4%; 95% CI: 76.0-80.8%) physicians. Jeans and expensive/fashionable clothing were broadly disapproved. For female physicians, the niqab/veil (756; 64.3%) and skirt (732; 62.2%) ranked highly alongside modest professional dress. Niqab preference varied significantly by age group (Kruskal-Wallis H = 14.93, p = 0.002), and female participants were more approving of the male national costume than their male counterparts (p = 0.01). Preferred physician attire was associated, across the board, with higher perceived respect (892; 75.9%), greater satisfaction (880; 74.8%), stronger follow-up intention (863; 73.4%), and better anticipated treatment compliance (773; 65.7%).

Conclusion: The white coat stands as the clearest, most cross-demographically consistent preference in this PHC population. Cultural modesty norms meaningfully shape how patients envision their female doctors' dress. Perhaps most importantly, how a physician looks appears to touch the very foundation of the therapeutic relationship, affecting willingness to return, comply with treatment, and engage openly. Dress code policies for PHCs in Riyadh should take these preferences seriously.

## Introduction

Long before evidence-based medicine became the organizing philosophy of clinical practice, ancient physicians understood that healing was partly a matter of perception. Hippocrates himself is said to have counselled that physicians should be clean in person, well dressed, and anointed with sweet-smelling unguents - a recognition, however early, that how a healer presents influences how they are received. In contemporary medicine, the physician-patient relationship remains the bedrock of effective care delivery. Patients form first impressions during the opening moments of a clinical encounter, drawing simultaneously on verbal cues - tone, empathy, clarity - and nonverbal ones, including the physician's body language, eye contact, and, notably, their clothing [1-3].

The white coat occupies a peculiar place in this dynamic. For well over a century, it has served as the defining uniform of the medical profession, carrying connotations of hygiene, scientific authority, and clinical competence. Yet it is not universally embraced. A strand of scholarship has long argued that the coat creates psychological distance - a visible symbol of hierarchy that may inhibit the kind of open, trusting conversation that good primary care depends upon. In parts of Scandinavia, primary care physicians rarely wear it; in Japan and Italy, it remains almost obligatory. The picture, then, is neither simple nor universal [4-8].

Within Saudi Arabia, the question of physician attire carries additional layers of meaning. Male physicians in the Kingdom may present in the traditional thobe (a full-length white robe) and shmagh or ghuttra (a head covering), in Western-style formal dress with a white coat, in surgical scrubs, or in various combinations thereof. Female physicians navigate a different set of choices: modest professional dress, the niqab (full face veil), long skirts, and various footwear options - all within a framework shaped by Islamic values of modesty and professionalism. These culturally specific garments simply do not appear in international attire research, making local data indispensable [9-11].

Saudi-based studies over the past decade have added meaningfully to our understanding, though with important gaps. The pioneering work by Al-Ghobain and colleagues in 2012 established that 62% of outpatient medical clinic attendees preferred formal attire and 85% wanted their physician in a white coat, with only one in 10 endorsing traditional Saudi dress [9].

Batais, working in a family medicine setting in Riyadh two years later, found a more nuanced picture: Western dress still led overall preferences, but patients were more willing to disclose sensitive personal matters, including sexual and psychological concerns, to a physician dressed in national Saudi attire. This context-dependency of attire preferences is a theme that recurs across the literature [10].

Al Amry and colleagues, in what remains the most directly comparable prior study, surveyed 443 patients in primary care clinics in Riyadh in 2018 and found white coat approval above 80%, with half of participants accepting traditional dress. They also showed that patients who valued physician appearance were significantly more likely to perceive a favorable doctor-patient relationship - an early indication that attire preferences are not merely aesthetic but clinically consequential [11].

On a larger scale, the online survey conducted by Basheikh et al. in 2020 reached 8,231 participants across Saudi Arabia and found surgical scrubs with a white coat to be the most preferred combination (39.3%), ahead of a Western shirt and tie with a white coat (30.3%). Traditional thobe with a white coat was selected by only 8.6% as a first preference, though this was a social media sample with a heavily educated, younger demographic [2].

The most recent Saudi contribution, a 2024 study examining surgeon attire, confirmed that female surgeons rated highest in a skirt, niqab, and white coat combination, while jeans and high heels, even paired with a white coat, attracted the lowest patient ratings. Over 71% of respondents agreed that surgeons should always wear white coats [12].

Internationally, the multi-country study by Houchens and colleagues (2022) surveyed over 9,000 patients across Italy, Japan, Switzerland, and the USA using a standardized instrument, finding broadly consistent preference for some form of white-coat attire, with scrubs favored specifically for surgeons and emergency physicians. The USA was distinctively more pro-formal than the European and Asian sites [8].

Against this backdrop, the Riyadh Second Health Cluster, with a total catchment population of approximately 3.8 million, represents one of the largest and most socially diverse primary care networks in the Kingdom. What its primary healthcare center (PHC) visitors think about physician dress has, to our knowledge, not been systematically studied. This matters: PHC visitors differ in important ways from hospitalized inpatients or specialist outpatient clinic attendees [1]. They come with different expectations, different relationship models, and different cultural compositions [9].

The present study therefore aimed, firstly, to document patient preferences for male and female physician attire among PHC visitors in this cluster; secondly, to determine how widely the perceived importance of physician appearance is shared across demographic groups; thirdly, to assess how attire preferences translate into perceptions of physician competence, professionalism, and respect; and fourthly, to explore the extent to which attire influences downstream relational outcomes - satisfaction, confidence, treatment compliance, and willingness to return for follow-up.

## Materials and methods

Study design and setting

This was a descriptive cross-sectional study carried out between the 1st of April and the 31st of May, 2026, in PHCs affiliated with the Riyadh Second Health Cluster, Ministry of Health, Saudi Arabia. The cluster serves a diverse population of roughly 3.8 million residents across urban and suburban communities and delivers primary care through family medicine clinics, general practice services, and urgent care facilities, making it one of the more demographically representative primary care networks in the region.

Study population and sampling

Any adult (aged ≥14 years) present in a PHC waiting area during data collection, whether attending as a patient or accompanying a patient, was eligible to participate. A convenience sample was used, given the practical realities of recruitment in a busy outpatient setting. Individuals who were acutely unwell, visibly distressed, or otherwise unable to engage with a paper questionnaire were not approached [11].

Sample size requirements were calculated using a single-proportion formula (assumed p = 0.50, 95% confidence, ±5% margin of error), adjusted for the finite target population of 3.8 million, yielding a minimum of 384 participants. We ultimately enrolled 1,176 - a sample yielding a margin of error of ±2.86%, well within acceptable limits for a community survey of this type.

The Questionnaire

The instrument was a bilingual (Arabic/English) self-administered questionnaire derived from validated tools used in prior Saudi and international attire studies, with modifications to incorporate Saudi-specific attire items [9-11].

It opened with an explanation of the study's purpose and a consent statement, followed by a demographic section (age, gender, marital status, education, employment, annual physician visit frequency). Participants then indicated their level of approval for a series of attire items worn by male physicians, including white medical lab coat, isotropic uniform, expensive or fashionable dress, surgical scrubs, jeans, a formal suit with shirt and tie, national costume (thobe and shmagh), and three types of footwear (slippers, sport boots, sandals), and, separately, for female physicians, including white coat, isotropic uniform, modest dress, expensive dress, surgical scrubs, jeans, niqab/veil, trousers, skirt, and five footwear categories (slipper shoes, sport boots, sandals, regular boots, low-heel shoes, and high heels).

The final section asked participants to rate, on the same five-point scale, how they felt about a physician who wears their preferred attire across 11 dimensions: five addressing perceived professional qualities (knowledge, experience, professionalism, credibility, respectfulness toward patients) and six addressing the doctor-patient relationship (general satisfaction, confidence, willingness to raise sensitive issues, desire to follow advice, treatment compliance intention, and intention to return for follow-up appointments). The scale ran from 1 (strongly disagree) to 5 (strongly agree), with a "don't care" option treated as a neutral midpoint (score 3). Arabic translation was validated by reverse translation and reviewed by two Saudi board-certified family medicine consultants [9-11].

Ethics

Ethical approval was obtained from the Research Ethics Committee of the Riyadh Second Health Cluster before the study commenced. Participation was voluntary and anonymous; no identifying information was collected.

Statistical analysis

Data were entered and analyzed in IBM SPSS Statistics, version 29 (IBM Corp., Armonk, NY). For each attire item, responses were categorized as "approved" (agree or strongly agree), "disapproved" (disagree or strongly disagree), or "neutral" (don't care). Approval rates are reported with 95% confidence intervals. For Likert-scale perception and relationship items, means, standard deviations, and 95% CIs are given; one-sample tests against the neutral midpoint (3.0) were used to assess whether scores differed significantly from neutrality. Chi-square tests examined associations between categorical approval and demographic variables; Mann-Whitney U tests compared two independent groups on ordinal attire ratings; Kruskal-Wallis tests handled three or more group comparisons. A two-tailed p < 0.05 was the threshold for statistical significance throughout [11].

## Results

Who participated

A total of 1,176 individuals completed the questionnaire - all of those approached who met eligibility criteria and agreed to participate. The sample skewed toward younger adults, with a mean age of 33.3 ± 9.8 years; nearly two-fifths (464; 39.5%) were between 14 and 29 years. Gender was nearly equal: 597 males (50.8%) and 579 females (49.2%). Most participants were married (824; 70.1%), held university-level or higher education (852; 72.4%), and were employed (769; 65.4%). The most common visit pattern was attending a physician more than five times a year (345; 29.3%), followed by two annual visits (266; 22.6%). Full demographic details appear in Table 1.

**Table 1:** Sociodemographic profile of study participants (N = 1,176). SD = standard deviation.

Variable	Category	n	%
Age group (years)	14-29	464	39.5
	30-39	386	32.8
	40-49	260	22.1
Mean ± SD = 33.3 ± 9.8 years	≥50	66	5.6
Gender	Male	597	50.8
	Female	579	49.2
Marital status	Married	824	70.1
	Single	352	29.9
Education	University and above	852	72.4
	Middle school	324	27.6
Employment	Employed	769	65.4
	Unemployed	395	33.6
Physician visits per year	1	149	12.7
	2	266	22.6
	3	165	14
	4	106	9
	5	133	11.3
	>5	345	29.3

How important is physician appearance?

The short answer, for this population, is: very. Exactly 950 participants (80.8%; 95% CI: 78.5-83.0%) agreed or strongly agreed with the statement that physician appearance matters to them, with 420 (35.7%) in the "strongly agree" category and 530 (45.1%) simply "agreeing." Fewer than one in eight participants actively disagreed (95; 8.1% disagree, 42; 3.6% strongly disagree). What is perhaps more striking is that this response was remarkably uniform: no significant differences were found by gender (χ² = 0.730, p = 0.393), marital status (χ² = 0.019, p = 0.890), education level (χ² = 0.016, p = 0.899), employment status (χ² = 0.496, p = 0.481), or age group (χ² = 0.952, p = 0.813). This is not a preference belonging to one demographic segment; it is, within this sample, a shared value [9,11].

What male physician attire do patients prefer?

Of all attire options presented for male physicians, the white medical lab coat attracted the strongest and broadest approval: 1,005 (85.5%; 95% CI: 83.4-87.5%). This placed it some 15 percentage points ahead of the next most approved item, the isotropic uniform (833; 70.8%; 95% CI: 68.2-73.4%). Surgical scrubs followed at 664 (56.5%; 95% CI: 53.6-59.3%), and sport boots - the only footwear item to receive majority approval - came in at 699 (59.4%). The national costume (thobe and shmagh) was approved by roughly half of respondents (579; 49.2%; 95% CI: 46.4-52.1%), and the formal suit with shirt and tie by 519 (44.1%) [9-11].

At the other end of the scale, expensive or fashionable dress was disapproved by 640 (54.4%) of participants - the highest disapproval rate of any male attire item. Jeans attracted disapproval from 585 (49.7%). Slipper shoes and sandals were each rejected by 530 (45.1%). These findings suggest that patients in this setting have fairly clear ideas about what they do not want to see, as well as what they prefer. Full data appear in Table 2.

**Table 2:** Approval rates for male physician attire items (N = 1,176). Rank based on approval proportion, highest to lowest. 95% CI = 95% confidence interval.

Attire item	Approve, n (%)	Disapprove, n (%)	Neutral, n (%)	95% CI (approve)	Rank
White medical lab coat	1005 (85.5%)	102 (8.7%)	69 (5.9%)	83.4-87.5%	1
Isotropic uniform	833 (70.8%)	192 (16.3%)	151 (12.8%)	68.2-73.4%	2
Sport boots	699 (59.4%)	292 (24.8%)	185 (15.7%)	56.6-62.2%	3
Surgical scrubs	664 (56.5%)	302 (25.7%)	210 (17.9%)	53.6-59.3%	4
National costume (Thobe & Shmagh)	579 (49.2%)	363 (30.9%)	234 (19.9%)	46.4-52.1%	5
Suit with shirt and tie	519 (44.1%)	393 (33.4%)	264 (22.4%)	41.3-47.0%	6
Sandals	326 (27.7%)	530 (45.1%)	320 (27.2%)	25.1-30.4%	7
Slipper shoes	315 (26.8%)	530 (45.1%)	331 (28.1%)	24.2-29.4%	8
Jeans	195 (16.6%)	585 (49.7%)	396 (33.7%)	14.5-18.7%	9
Expensive/fashionable dress	132 (11.2%)	640 (54.4%)	404 (34.4%)	9.4-13.1%	10

Female physician attire preferences

Preference patterns for female physician attire shared some features with those for male physicians - the white coat leading comfortably, the isotropic uniform in second - but diverged in ways that reflect the culturally specific expectations patients hold for female doctors in Saudi Arabia [9,11,12].

The white coat attracted 922 (78.4%) approvals (95% CI: 76.0-80.8%). The isotropic uniform followed at 834 (70.9%). After that, modest attire options dominated: the niqab/veil received 756 (64.3%) approvals (95% CI: 61.5-67.0%), the skirt 732 (62.2%; 95% CI: 59.5-65.0%), and the modest dress received 714 (60.7%). Surgical scrubs, often associated with clinical competence in Saudi and regional research, were approved by 646 (54.9%). At the bottom, trousers attracted only 171 (14.5%) approvals, jeans 141 (12.0%), and high-heel shoes a mere 74 (6.3%), with the latter also posting the study's highest single disapproval rate at 665 (56.5%). Table 3 provides the complete breakdown.

**Table 3:** Approval rates for female physician attire items (N = 1,176). Rank based on approval proportion, highest to lowest. 95% CI = 95% confidence interval.

Attire item	Approve, n (%)	Disapprove, n (%)	Neutral, n (%)	95% CI (approve)	Rank
White medical lab coat	922 (78.4%)	155 (13.2%)	99 (8.4%)	76.0-80.8%	1
Isotropic uniform	834 (70.9%)	201 (17.1%)	141 (12.0%)	68.3-73.5%	2
Niqab/veil	756 (64.3%)	260 (22.1%)	160 (13.6%)	61.5-67.0%	3
Skirt	732 (62.2%)	264 (22.4%)	180 (15.3%)	59.5-65.0%	4
Modest dress	714 (60.7%)	260 (22.1%)	202 (17.2%)	57.9-63.5%	5
Sport boots	676 (57.5%)	292 (24.8%)	208 (17.7%)	54.6-60.3%	6
Surgical scrubs	646 (54.9%)	321 (27.3%)	209 (17.8%)	52.1-57.8%	7
Low heel shoes	634 (53.9%)	337 (28.7%)	205 (17.4%)	51.1-56.7%	8
Regular shoes/boots	561 (47.7%)	188 (16.0%)	415 (35.3%)	44.9-50.6%	9
Sandals	433 (36.8%)	424 (36.1%)	319 (27.1%)	34.1-39.6%	10
Trousers	171 (14.5%)	623 (53.0%)	382 (32.5%)	12.5-16.6%	11
Jeans	141 (12.0%)	620 (52.7%)	415 (35.3%)	10.2-13.8%	12
Expensive/fashionable dress	133 (11.3%)	600 (51.0%)	443 (37.7%)	9.5-13.1%	13
High heel shoes	74 (6.3%)	665 (56.5%)	437 (37.2%)	5.0-7.7%	14

Do demographics shape preferences?

For most attire items, including the white coat, surgical scrubs, jeans, skirt, and niqab when analyzed by gender, the answer was no. The white coat's dominance held steady regardless of how the sample was sliced, with no significant differences by gender, age, marital status, education, or occupation (all p > 0.05 for both male and female physician white coat approval). This demographic near-uniformity is one of the more notable findings of the study [9,11].

Two exceptions are worth noting. Female participants were more approving of the male national costume (300; 51.8%) than male participants (279; 46.7%), a difference that reached statistical significance on the Mann-Whitney U test (U = 158,202; p = 0.01). Niqab/veil approval for female physicians varied meaningfully across age groups (Kruskal-Wallis H = 14.93, df = 3, p = 0.002): the oldest group (≥50 years) averaged a mean score of 3.09 on the five-point scale, noticeably lower than the 14-29 (3.70), 30-39 (3.62), and 40-49 (3.66) groups. Table 4 summarizes the inferential findings.

**Table 4:** Inferential tests for selected attire items by demographic variable. * p < 0.05; ** p < 0.01; ns = not significant; MD = medical doctor; mean scores on a five-point Likert scale.

Attire item	Demographic	Test	Statistic	p-value	Finding
White coat (male MD)	Gender	Chi-square	χ² = 0.047	0.829	No difference (M = 512, 85.8% vs. F = 493, 85.1%)
White coat (male MD)	Age group	Kruskal-Wallis	H = 1.334	0.721	No difference (range = 83.9-89.4%)
White coat (male MD)	Education	Chi-square	χ² = 0.000	1.000	Identical (University = 728, 85.4% vs. Middle = 277, 85.5%)
National costume (male MD)	Gender	Mann-Whitney U	U = 158,202	0.010*	Females more approving (300, 51.8% vs. 279, 46.7%)
Niqab/veil (female MD)	Age group	Kruskal-Wallis	H = 14.927	0.002**	≥50 years lower (mean = 3.09 vs. 3.62-3.70)
Niqab/veil (female MD)	Gender	Chi-square	χ² = 0.160	0.689	No difference (F = 376, 64.9% vs. M = 380, 63.7%)
Skirt (female MD)	Gender	Chi-square	χ² = 0.950	0.330	No difference (F = 369, 63.7% vs. M = 363, 60.8%)
Importance of appearance	All variables	Chi-square	All ns	All > 0.05	No associations with any demographic

What does attire do to the doctor-patient relationship?

When asked how a physician wearing their preferred attire made them feel, participants were consistently and significantly positive across all 16 outcome items - five relating to professional perception and six to the relational quality of the encounter. Table 5 covers the perception items, and Table 6 covers the relationship items [1,2,10].

**Table 5:** Impact of preferred attire on perceived professional qualities (N = 1,176). Scores on a five-point scale (1 = strongly disagree, 5 = strongly agree); p vs. neutral = one-sample test against midpoint 3.0.

Perception item	Agree/strongly agree, n (%)	Disagree, n (%)	Mean (SD)	95% CI	p vs. neutral
More respectful to patients	892 (75.9%)	168 (14.3%)	3.93 (1.14)	3.87-4.00	<0.001
More credible	772 (65.6%)	233 (19.8%)	3.67 (1.20)	3.60-3.74	<0.001
More professional & efficient	762 (64.8%)	242 (20.6%)	3.67 (1.24)	3.59-3.74	<0.001
More knowledgeable	747 (63.5%)	275 (23.4%)	3.62 (1.26)	3.54-3.69	<0.001
More experienced & mature	710 (60.4%)	112 (9.5%)	3.83 (1.05)	3.77-3.89	<0.001
Composite score	—	—	3.74 (0.51)	3.71-3.77	<0.001

**Table 6:** Impact of preferred attire on the doctor-patient relationship (N = 1,176). Scores on a five-point scale (1 = strongly disagree, 5 = strongly agree); p vs. neutral = one-sample test against midpoint 3.0.

Relationship item	Agree/strongly agree, n (%)	Disagree, n (%)	Mean (SD)	95% CI	p vs. neutral
General satisfaction	880 (74.8%)	164 (13.9%)	3.88 (1.10)	3.82-3.94	<0.001
Desire to follow up	863 (73.4%)	205 (17.4%)	3.81 (1.18)	3.75-3.88	<0.001
Confidence in dealing	819 (69.6%)	210 (17.9%)	3.75 (1.18)	3.69-3.82	<0.001
Desire to be compliant	773 (65.7%)	240 (20.4%)	3.69 (1.22)	3.62-3.76	<0.001
Desire to follow advice	719 (61.1%)	279 (23.7%)	3.57 (1.26)	3.50-3.64	<0.001
More open about sensitive issues	691 (58.8%)	294 (25.0%)	3.51 (1.28)	3.44-3.58	<0.001
Composite score	—	—	3.70 (0.49)	3.67-3.73	<0.001

On the perception side, three in four participants (892; 75.9%; 95% CI: 73.4-78.3%) agreed that their preferred attire made the physician seem more respectful to patients - the highest-ranked item in this group. More credible (772; 65.6%), more professional and efficient (762; 64.8%), more knowledgeable (747; 63.5%), and more experienced and mature (710; 60.4%) rounded out the list. The composite perception score across all five items was 3.74 ± 0.51 (95% CI: 3.71-3.77), solidly above the neutral midpoint of 3 (p < 0.001).

Regarding the relational outcomes, general satisfaction attracted the most agreement (880; 74.8%; 95% CI: 72.3-77.3%), just ahead of follow-up intention (863; 73.4%; 95% CI: 70.9-75.9%) and confidence in dealing with the physician (819; 69.6%). Treatment compliance intention was positive in 773 (65.7%) of respondents, willingness to follow specific advice in 719 (61.1%), and willingness to raise sensitive issues in 691 (58.8%) - still a majority, though the narrowest of the six. The composite relationship score was 3.70 ± 0.49 (95% CI: 3.67-3.73), again comfortably above neutrality (p < 0.001) (Table 6) [10,11].

## Discussion

Taken together, the findings from this study point in a broadly coherent direction: PHC visitors in Riyadh hold strong, clear, and demographically consistent preferences for how their doctors dress - preferences that go well beyond aesthetics and touch on some of the most clinically important features of primary care, including trust, compliance, and return for follow-up [8,9,11].

The white coat - A persistent symbol

The white coat's dominance in this sample is unambiguous. Its 1,005 (85.5%) approval ratings for male physicians and 922 (78.4%) for female physicians are not only high in absolute terms but are also essentially invariant across all demographic subgroups - age, gender, education, marital status, and employment. Few findings in cross-sectional survey research achieve this kind of demographic stability, and it deserves emphasis [9,11].

Our figure sits close to what Al-Ghobain et al. found in hospital outpatient clinics in 2012 (85%), and is higher than the 47% who, in that same study, preferred formal attire in the general sense, suggesting that the white coat carries meaning that formal dress alone does not [9].

Al Amry and colleagues, studying primary care in Riyadh a few years later, found white coat approval at over 80%, and explicitly linked patients who valued physician appearance with a positive perception of the doctor-patient relationship. Our data extend that observation: the relationship between appearance-valuing and relational outcomes appears not just to exist but to operate with some force across all 11 dimensions we measured [11].

The same picture emerges from Basheikh et al.'s 2020 national online survey, where the common thread across all preferred combinations - scrubs with white coat, shirt and tie with white coat - was precisely the presence of the white coat. Houchens and colleagues, surveying patients across Europe, Asia, and North America, found that 78.6% of over 9,000 participants preferred at least one form of attire that included a white coat [2,8].

All of this points to a straightforward practical implication: making the white coat a required element of PHC physician dress in this cluster would meet the preferences of the large majority of patients, regardless of what those patients look like demographically.

The national costume - A primary care effect?

The 49.2% approval rate for the thobe and shmagh (national costume) for male physicians deserves some unpacking, because it sits markedly higher than the 9.7% recorded in Al-Ghobain et al.'s hospital outpatient study, higher than the 26.3% found by Batais in a family medicine center, and roughly in line with the 47% from Al Amry et al.'s primary care study [9-11].

A plausible interpretation is that the clinical setting itself shapes cultural expectations. In hospital specialist clinics, the model of the physician is fundamentally that of a technical expert, and Western professional dress seems to match that expectation. In primary care, where relationship continuity, cultural familiarity, and holistic understanding matter more, traditional dress may signal something patients actively value: cultural proximity and accessibility [10].

The gender difference we found within this item (female participants approving more than males; p = 0.01) adds texture here. Batais noted that patients of both sexes were more willing to raise sensitive psychosocial and sexual matters with a traditionally dressed physician. Our finding suggests that female patients may be especially receptive to this cultural signal, perhaps because traditional dress in a male physician conveys a form of shared social conservatism that makes intimate disclosures feel safer [10].

Female physician attire - Cultural modesty as professionalism

The preference data for female physician attire reflect what one might call a patient expectation of culturally anchored professionalism. The white coat ranks first, but the niqab/veil (756; 64.3%), skirt (732; 62.2%), and modest dress (714; 60.7%) rank well above scrubs - a reversal of what tends to be found in Western settings, where surgical scrubs are increasingly the default across genders [9,12].

The 2024 surgeon attire study by Aloraini and colleagues found the same hierarchy: female surgeons rated highest in the skirt, niqab, and white coat combination, and lowest when dressed in jeans or high heels, even with a white coat. Our PHC data largely replicate this pattern, suggesting it is not specialty-specific but reflective of a broader Saudi patient preference for female physicians who present within recognizable norms of Islamic modesty [12].

High heel shoes received the lowest approval of any item in the entire study (74; 6.3%) and the highest single disapproval rate (665; 56.5%). In a clinical context where trust and perceived hygiene are partly read through visual cues, footwear that signals fashion rather than function appears to create genuinely negative impressions. This is consistent with Aloraini et al.'s finding that heeled shoes significantly depressed patient ratings across trust, care, approachability, and comfort domains [12].

The age-group variation in niqab approval (p = 0.002) is perhaps the most intriguing finding in the female attire section. Older patients (≥50 years) were less approving of the niqab for their female doctors than younger respondents - a result that runs counter to the intuitive assumption that more traditionally minded older patients would most strongly prefer traditional Islamic dress. One speculative explanation is that older patients may have formed their medical expectations in an era when female physicians dressed more uniformly in professional Western attire, and that younger patients have grown up in an environment where the niqab in professional life is more normalized, even desirable. This warrants follow-up with qualitative methods [12].

Attire and the architecture of the doctor-patient relationship

Perhaps the most clinically significant thread running through this study is the consistent, statistically robust association between perceived physician appearance and a range of outcomes that sit at the heart of effective primary care: trust, openness, treatment adherence, and continuity [1,2].

The finding that 863 (73.4%) of participants reported a greater intention to return for follow-up when their physician was appropriately dressed, and that 773 (65.7%) reported an increased intention to comply, is worth pausing on. In a primary care context, where the management of hypertension, diabetes, and other chronic conditions depends entirely on patients returning, engaging, and taking their medications, these are not trivial percentages. They suggest that attire is not merely a first-impression variable but may carry influence across the trajectory of care [10].

Rehman et al.'s foundational 2005 study, which arguably launched the modern era of physician attire research, found exactly this: professionally dressed doctors elicited more trust and a better self-reported likelihood of following advice. Over 20 years later, in a culturally distinctive PHC setting in Saudi Arabia, the pattern holds [1].

Willingness to discuss sensitive issues - the relationship item with the lowest agreement (691; 58.8%) - is worth examining in light of findings from Batais, who showed that patients in his Saudi family medicine sample were distinctly more willing to raise psychosocial, sexual, and psychological concerns with a physician in national Saudi dress than with one in Western attire. Our data do not allow us to dissect that particular contrast since we measured willingness across all preferred attire types, but the implication is there: optimal attire for a sensitive consultation may not be the same as optimal attire for a routine encounter. This is a refinement worth building into future attire research and clinical guidelines [10].

Limitations

A few limitations should be acknowledged. Convenience sampling within waiting areas means that those who accessed PHC services during our data collection window are represented; those who do not use PHCs, or who attended at different times, may hold different views. The absence of attire photographs is a real constraint: verbal descriptions of attire leave room for variation in how participants imagined each item. The cross-sectional design means we can speak to associations but not causal pathways. And while the sample of 1,176 substantially exceeds our calculated minimum, it remains a sample from one health cluster, and conclusions should be applied to other Saudi regions with appropriate caution.

## Conclusions

Three things, above all, stand out from this study. First, the white medical lab coat is the single clearest and most widely shared preference for PHC physician attire in Riyadh, a preference that does not meaningfully vary across age, gender, education, or any other demographic variable we examined. Second, for female physicians, cultural modesty attire (niqab/veil, skirt, modest dress) sits alongside the white coat as a positively valued, widely endorsed choice, with high heels and jeans as clearly unwelcome at the other end of the spectrum. Third, and arguably most important for healthcare planners, how a physician dresses appears to matter for outcomes that genuinely affect care quality: patients reported a greater likelihood of following advice, attending follow-up appointments, and engaging with treatment when their physician wore their preferred attire.

Taken together, these findings make a reasonably strong case for formalizing PHC dress code policies in the Riyadh Second Health Cluster, policies that anchor on the white coat as a mandatory element, allow culturally appropriate modest attire for female physicians, and steer away from casual or fashion-oriented dress. Future work should use photographic stimuli to sharpen preference measurement, examine how attire preferences interact with consultation type (routine versus sensitive), and explore physician perspectives on dress code feasibility.

## Appendices

**Table 7:** Data collection tool (questionnaire).

No.	Section	Question (Arabic)	Question (English)	Response options / Scale
	Section 1 – Consent			
	Consent	هل توافق على اجراء هذا الاستبيان ؟	Do you agree to take part in this survey?	أوافق/Accept — لا أوافق/I refuse
	Section 2 – Demographic Information			
1	Demographics	العمر	Age	18 to 75, or Older than 75 (single selection)
2	Demographics	الجنس	Sex	ذكر/Male — أنثى/Female
3	Demographics	الحالة الإجتماعية	Marital status	أعزب/Single — متزوج/Married — أرمل/Widowed — مطلق/Divorced
4	Demographics	المستوى التعليمي	Level of education	أمي/Illiterate — ابتدائي/Elementary — متوسط/Intermediate — ثانوي/Secondary — جامعي فأعلى/University and higher
5	Demographics	طبيعة العمل	Nature of work	يعمل/Employed — لا يعمل/Unemployed
6	Demographics	عدد مرات زيارة الطبيب في السنة	Number of doctor visits per year	1 — 2 — 3 — 4 — 5 — أكثر من 5/More than 5
7	Demographics	مظهر الطبيب مهم جدًا بالنسبة لي	The doctor's appearance is very important to me	Strongly agree / Agree / I don't care / Disagree / Strongly disagree
	Section 3 – Opinion on Male Doctor's Attire			
8	Male doctor attire	المعطف الطبي الأبيض	What do you think about a doctor wearing: White medical lab coat?	Strongly agree / Agree / I don't care / Disagree / Strongly disagree
9	Male doctor attire	الزي الرسمي الموحد	What do you think about a doctor wearing: Isotropic/uniform dressing?	Strongly agree / Agree / I don't care / Disagree / Strongly disagree
10	Male doctor attire	اللباس الغالي والأنيق	What do you think about a doctor wearing: Expensive & elegant clothing?	Strongly agree / Agree / I don't care / Disagree / Strongly disagree
11	Male doctor attire	اللباس الجراحي	What do you think about a doctor wearing: Surgical dressing?	Strongly agree / Agree / I don't care / Disagree / Strongly disagree
12	Male doctor attire	الجينز	What do you think about a doctor wearing: Jeans?	Strongly agree / Agree / I don't care / Disagree / Strongly disagree
13	Male doctor attire	البدلة (قميص، بنطال وربطة عنق)	What do you think about a doctor wearing: Suit (shirt, trousers and tie)?	Strongly agree / Agree / I don't care / Disagree / Strongly disagree
14	Male doctor attire	اللباس الوطني (الثوب والشماغ)	What do you think about a doctor wearing: National costume (Thoub & Shmagh)?	Strongly agree / Agree / I don't care / Disagree / Strongly disagree
15	Male doctor attire	الأحذية المفتوحة	What do you think about a doctor wearing: Slipper shoes?	Strongly agree / Agree / I don't care / Disagree / Strongly disagree
16	Male doctor attire	الأحذية الرياضية	What do you think about a doctor wearing: Sport shoes?	Strongly agree / Agree / I don't care / Disagree / Strongly disagree
17	Male doctor attire	الصندل	What do you think about a doctor wearing: Sandals?	Strongly agree / Agree / I don't care / Disagree / Strongly disagree
	Section 4 – Opinion on Female Doctor's Attire			
18	Female doctor attire	المعطف الطبي الأبيض	What do you think about a female doctor wearing: White medical lab coat?	Strongly agree / Agree / I don't care / Disagree / Strongly disagree
19	Female doctor attire	الزي الرسمي الموحد	What do you think about a female doctor wearing: Isotropic/uniform dressing?	Strongly agree / Agree / I don't care / Disagree / Strongly disagree
20	Female doctor attire	اللباس المحتشم	What do you think about a female doctor wearing: Modest dress?	Strongly agree / Agree / I don't care / Disagree / Strongly disagree
21	Female doctor attire	اللباس الغالي والأنيق	What do you think about a female doctor wearing: Expensive & elegant clothing?	Strongly agree / Agree / I don't care / Disagree / Strongly disagree
22	Female doctor attire	اللباس الجراحي	What do you think about a female doctor wearing: Surgical dressing?	Strongly agree / Agree / I don't care / Disagree / Strongly disagree
23	Female doctor attire	الجينز	What do you think about a female doctor wearing: Jeans?	Strongly agree / Agree / I don't care / Disagree / Strongly disagree
24	Female doctor attire	النقاب	What do you think about a female doctor wearing: Niqab/veil?	Strongly agree / Agree / I don't care / Disagree / Strongly disagree
25	Female doctor attire	البنطال	What do you think about a female doctor wearing: Trousers?	Strongly agree / Agree / I don't care / Disagree / Strongly disagree
26	Female doctor attire	التنورة	What do you think about a female doctor wearing: Skirt?	Strongly agree / Agree / I don't care / Disagree / Strongly disagree
27	Female doctor attire	الأحذية المفتوحة	What do you think about a female doctor wearing: Slipper shoes?	Strongly agree / Agree / I don't care / Disagree / Strongly disagree
28	Female doctor attire	الأحذية الرياضية	What do you think about a female doctor wearing: Sport shoes?	Strongly agree / Agree / I don't care / Disagree / Strongly disagree
29	Female doctor attire	الصندل	What do you think about a female doctor wearing: Sandals?	Strongly agree / Agree / I don't care / Disagree / Strongly disagree
30	Female doctor attire	الأحذية العادية (البوت)	What do you think about a female doctor wearing: Usual shoes (boots)?	Strongly agree / Agree / I don't care / Disagree / Strongly disagree
31	Female doctor attire	الأحذية ذات الكعب المنخفض	What do you think about a female doctor wearing: Low-heel women's shoes?	Strongly agree / Agree / I don't care / Disagree / Strongly disagree
32	Female doctor attire	الأحذية ذات الكعب العالي	What do you think about a female doctor wearing: High-heel women's shoes?	Strongly agree / Agree / I don't care / Disagree / Strongly disagree
	Section 5 – Perception of Doctor Wearing Preferred Attire			
33	Perception of doctor	أكثر معرفة ومعلومات	I feel the doctor who wears my preferred attire is: More knowledge and has more information	Strongly agree / Agree / I don't care / Disagree / Strongly disagree
34	Perception of doctor	أكثر خبرة ونضجًا	I feel the doctor who wears my preferred attire is: More experienced and mature	Strongly agree / Agree / I don't care / Disagree / Strongly disagree
35	Perception of doctor	أكثر مهنية وكفاءة	I feel the doctor who wears my preferred attire is: More professional and efficient	Strongly agree / Agree / I don't care / Disagree / Strongly disagree
36	Perception of doctor	أكثر مصداقية	I feel the doctor who wears my preferred attire is: More credible	Strongly agree / Agree / I don't care / Disagree / Strongly disagree
37	Perception of doctor	أكثر احترامًا لمرضاه	I feel the doctor who wears my preferred attire is: More respectful to patients	Strongly agree / Agree / I don't care / Disagree / Strongly disagree
	Section 6 – Doctor–Patient Relationship			
38	Doctor-patient relationship	رضا عام	When I consult a doctor wearing my preferred attire, I feel: General satisfaction	Strongly agree / Agree / I don't care / Disagree / Strongly disagree
39	Doctor-patient relationship	ثقة في التعامل	When I consult a doctor wearing my preferred attire, I feel: Confidence in dealing with the doctor	Strongly agree / Agree / I don't care / Disagree / Strongly disagree
40	Doctor-patient relationship	جرأة أكثر في طرح القضايا الحساسة	When I consult a doctor wearing my preferred attire, I feel: More daring in presenting sensitive issues	Strongly agree / Agree / I don't care / Disagree / Strongly disagree
41	Doctor-patient relationship	رغبة في اتباع نصائحه	When I consult a doctor wearing my preferred attire, I feel: Desire to follow his advice	Strongly agree / Agree / I don't care / Disagree / Strongly disagree
42	Doctor-patient relationship	رغبة في الالتزام بتعليماته	When I consult a doctor wearing my preferred attire, I feel: Desire to comply with his instructions	Strongly agree / Agree / I don't care / Disagree / Strongly disagree
43	Doctor-patient relationship	رغبة في المتابعة معه في الزيارات القادمة	When I consult a doctor wearing my preferred attire, I feel: Desire to follow up with him in future visits	Strongly agree / Agree / I don't care / Disagree / Strongly disagree
